# Post-mortem imaging of the infant and perinatal dura mater and superior sagittal sinus using optical coherence tomography

**DOI:** 10.1007/s00414-017-1570-1

**Published:** 2017-04-07

**Authors:** Emma C. Cheshire, Roger D. G. Malcomson, Shiju Joseph, Asif Adnan, David Adlam, Guy N. Rutty

**Affiliations:** 1 East Midlands Forensic Pathology Unit, University of Leicester, Robert Kilpatrick Building, Level 3 Leicester Royal Infirmary, Leicester, LE2 7LX UK; 2 Histopathology Department, Leicester Royal Infirmary, Infirmary Close, Leicester, LE1 5WW UK; 3 Department of Cardiovascular Sciences, Glenfield Hospital, Groby Road, Leicester, LE3 9QP UK

**Keywords:** Post-mortem, Optical coherence tomography, Dura mater, Infant, Perinatal, Superior sagittal sinus

## Abstract

Infants and young children are likely to present with subdural haemorrhage (SDH) if they are the victims of abusive head trauma. In these cases, the most accepted theory for the source of bleeding is the bridging veins traversing from the surface of the brain to the dura mater. However, some have suggested that SDH may result from leakage of blood from a dural vascular plexus. As post-mortem examination of the bridging veins and dura is challenging, and imaging modalities such as magnetic resonance and computed tomography do not have the resolution capabilities to image small blood vessels, we have trialled the use of intravascular and benchtop optical coherence tomography (OCT) systems for imaging from within the superior sagittal sinus (SSS) and through the dura during five infant/perinatal autopsies. Numerous vessel-like structures were identified using both OCT systems. Measurements taken with the intravascular rotational system indicate that the approximate median diameters of blood vessels entering anterior and posterior segments of the SSS were 110 μm (range 70 to 670 μm, *n* = 21) and 125 μm (range 70 to 740 μm, *n* = 23), respectively. For blood vessels close to the wall of the SSS, the median diameters for anterior and posterior segments of the SSS were 80 μm (range 40 to 170 μm, *n* = 25) and 90 μm (range 30 to 150 μm), respectively. Detailed characterisation of the dural vasculature is important to aid understanding of the source of SDH. High resolution 3-dimensional reconstructions of the infant dural vasculature may be possible with further development of OCT systems.

## Introduction

Subdural haemorrhage is a common finding in abusive head trauma (AHT), with this type of bleeding reported in 80–92% of cases reported in both prospective studies [1–3] and retrospective reviews of medical, autopsy and legal reports [4, 5]. Historically, the most commonly suggested source of subdural bleeding is the traumatic damage of bridging veins which traverse from the surface of the brain to the dura mater [6–8]. However, several alternative hypotheses for the source of subdural bleeding have seen suggested over the last 2 decades, including the leakage of blood from a dense vascular plexus within the dural membrane [9, 10]. Ruptured bridging veins are also challenging to demonstrate on imaging (such as magnetic resonance (MR) and computed tomography (CT)) due to resolution limitations and also during autopsies due to disruption consequent of opening the cranium. One of the few reported demonstrations of ruptured bridging veins was in a study in which barium sulphate was injected into the superior sagittal sinus (SSS), and extravasation of contrast agent into the surrounding tissue was detected by x-ray [11].

**Fig. 1:**
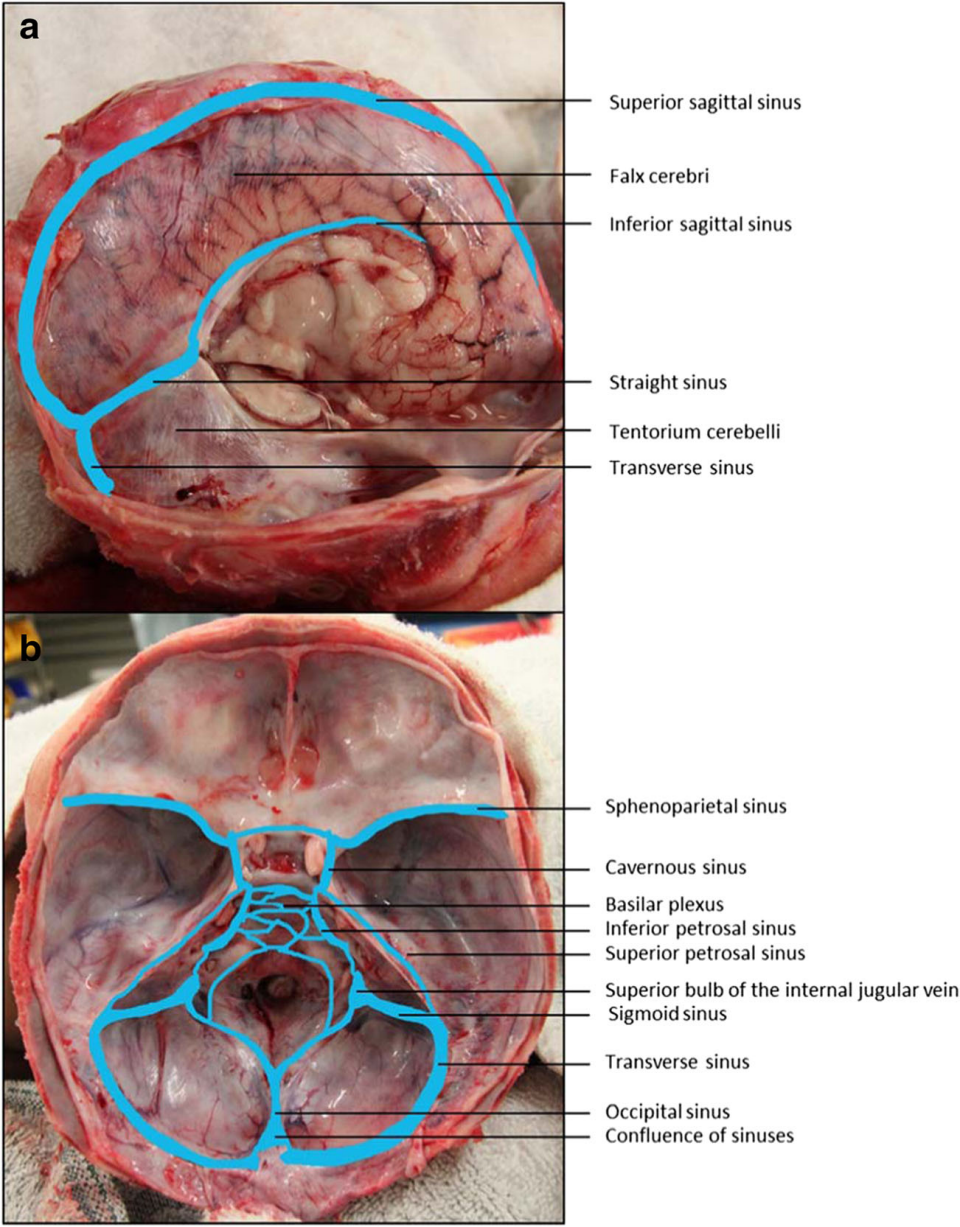
The dural venous sinuses. **a** View of the falx cerebri and tentorium cerebelli after right hemisperectomy. **b** Cranial base after removal of the brain

The larger blood vessels and drainage channels associated with the dural membrane include the dural venous sinuses (Fig. 1), which drain blood from the brain to the jugular veins and the arteries which supply blood to the dura. The dural venous sinuses run between the outer, or endosteal, layer of the dura mater and the inner, meningeal layer (with the exception of the inferior sagittal sinus which lies between the folds of the free margin of the falx cerebri and tentorium cerebelli) [12, 13]. The major blood supply to the dura comes from the middle meningeal artery, with the ascending pharyngeal, internal carotid, maxillary, vertebral and occipital arteries also carrying blood to the dura to a lesser extent. Accompanying the branches of the middle meningeal artery, which travel predominately in the endosteal layer of the dura despite their name, are branches of the middle meningeal vein which also travel in this layer [13–15]. The bridging veins are the terminal sections of cerebral veins which traverse from the surface of the brain though the leptomeninges to the dural venous sinuses, either directly emptying into a sinus or by travelling in association with the dural membrane to the nearest sinus.

The meningeal layer of the dura mater is often described as dense, strong and fibrous, with blood vessels mainly described in the endosteal layer. Although not often alluded to in the current literature, the dura mater has been described in the past studies as being a highly vascular tissue [16–18] with the suggestion of an extremely rich capillary network on the inner dural surface [17–19].

Morphological assessments of the bridging veins and vessels within the dura are challenging, particularly in infants and neonates. In this age group, the calvarial suture lines are unossified and are in fibrous continuity with the underlying dura and the fontanelles [20]. Due to these attachments, the infant skull bones cannot be removed as a cap, as would occur in an adult post-mortem. Although there are methods of opening the infant calvarium which leave a midline strip of bone overlying the SSS [21], it is still necessary to use scissors (or special “infant skull shears”) or an oscillating autopsy saw, to cut through the bones [21, 22]. These methods are usually not only damaging to the dura but also will disrupt those bridging veins that do not directly join the SSS (manuscript in preparation). Furthermore, routine histological examination does not enable in situ evaluation of the blood vessels, and imaging systems such as MR and CT do not have the resolution capabilities to capture very small vessels. Improved methods of visualising the vascular systems associated with the dura are needed to aid the evaluation of the source of subdural bleeding.

Optical coherence tomography (OCT) is an imaging modality that provides real-time, high-resolution (between 1 and 20 μm), sub-surface images of cell and tissue microstructure using near-infrared light [23–25]. This type of imaging is analogous to ultrasound B-mode imaging [23] except that, instead of using high frequency sound, an optical beam is directed at the tissue. One of its common uses is in ophthalmology for diagnostic imaging of the anterior eye and retina [26, 27] and more recently for the characterisation of coronary atherosclerosis using intravascular OCT systems [28]. Optical coherence tomography uses interferometry to derive information about depth and reflectivity from backscattered light (i.e. the amount of light reflected back from the various components within the tissue of interest) and can produce both 2- and 3-dimensional data sets [25].

Optical coherence tomography has recently been translated into the post-mortem setting for the purpose of providing virtual histology of the coronary arteries as a minimally invasive autopsy technique [24]. In this paper, we investigate the potential use of OCT for post-mortem imaging within the SSS and through the dura mater to the structures closely underlying this membrane, using an intravascular and benchtop system, respectively, in several proofs of concept studies.

## Materials and methods

### Case selection

Parental consent was gained for OCT in two infants (an 8 and a 9-week-old male) and three perinatal (two 1-day-old females and a 3-day-old male) autopsies undertaken at Leicester between September 2014 and March 2015 as part of a regional paediatric autopsy service. Owing to the limited availability of the benchtop system, all five cases had rotational OCT, whereas only two cases had benchtop OCT.

### Imaging systems

A rotational clinical OCT system, the St. Jude Ilumien^TM^ Optis^TM^ (St. Jude Medical Inc., USA), was used to image within the SSS. This intravascular imaging modality uses a catheter with fibre-optic technology that emits near-infrared light.

A clinical Fourier domain benchtop system, the Bioptigen Envisu^TM^ C-class (Bioptigen, USA), was used to image the dural membrane. This system employs a non-contact mode of use with a handheld or mounted imaging probe. The anterior chamber imaging probe of this ophthalmological OCT system was used for acquiring images.

### Rotational optical coherence tomography

**Fig. 2:**
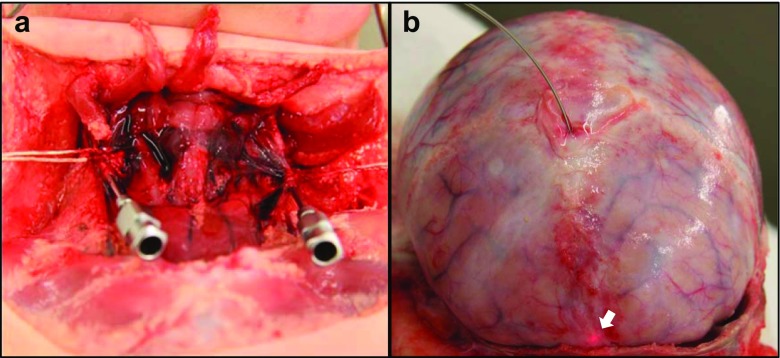
Rotational OCT setup. **a** Dissection and cannulation of the internal jugular veins. **b** Insertion of the catheter into the SSS through the anterior fontanelle with red light (*arrow*) showing the tip of the catheter

The calvarial bones were removed by a method previously described [29] to leave the dura mater intact. The internal jugular veins were dissected and cannulated with Tibbs cannulae (Fig. 2a) to allow perfusion of tap water into the dural venous sinuses to restore intraluminal pressure to aid imaging within the SSS. Only one jugular vein was perfused to allow drainage of excess water and to mitigate swelling of the brain owing to excess retrograde perfusion of the cerebral veins. A guide wire (0.014″ diameter with hydrophilic coating, Whisper MS by Abott Vascular) was passed through a needle and used to position a catheter (C7 Dragonfly, St. Jude Medical Inc., USA) through a hole in the anterior fontanelle (Seldinger technique) along the sinus to the location of imaging. The red light visible near the tip of the catheter could be used to assess the location of the catheter tip within the sinus (Fig. 2b). The imaging element of the catheter performs an automatic axial traversal (a “pullback”) to image a specified length (pullback at 10 mm/s for a length of 54 mm). To ensure all sections of the sinus were imaged, pullbacks were overlapped and repeated three times for each 54 mm section.

Due to the large amount of data retrieved from each pullback (541 frames/pullback), two videos of good quality (one from both an anterior and posterior section of the SSS) were selected from one case (1-day-old female) for detailed frame-by-frame analysis.

### Frame-by-frame analysis of rotational pullbacks

Each frame is a 2-dimensinal slice of a 3-dimensional structure; therefore, it is not possible to determine the exact orientation of the vessels close to, and entering, the SSS. For this reason, only approximate measurements of structures which appeared to be blood vessels due to their cylindrical shape, branching nature, or the fact they were directly entering the sinus, were made. Diameter measurements were made of vessel-like structures close to the wall of the SSS when they appeared to be imaged in a cross-sectional plane. As blood vessels entering the SSS would obviously not appear in cross section at the time of joining the sinus, the gap created in the wall of the sinus was measured to provide an indication of vessel size.

The mean diameters of both anterior and posterior segments of the SSS were also calculated, as was the cross-sectional area of both segments by manually delimiting the edge of the SSS wall in each frame using the manufacturer’s software tool provided.

### Benchtop optical coherence tomography

**Fig. 3:**
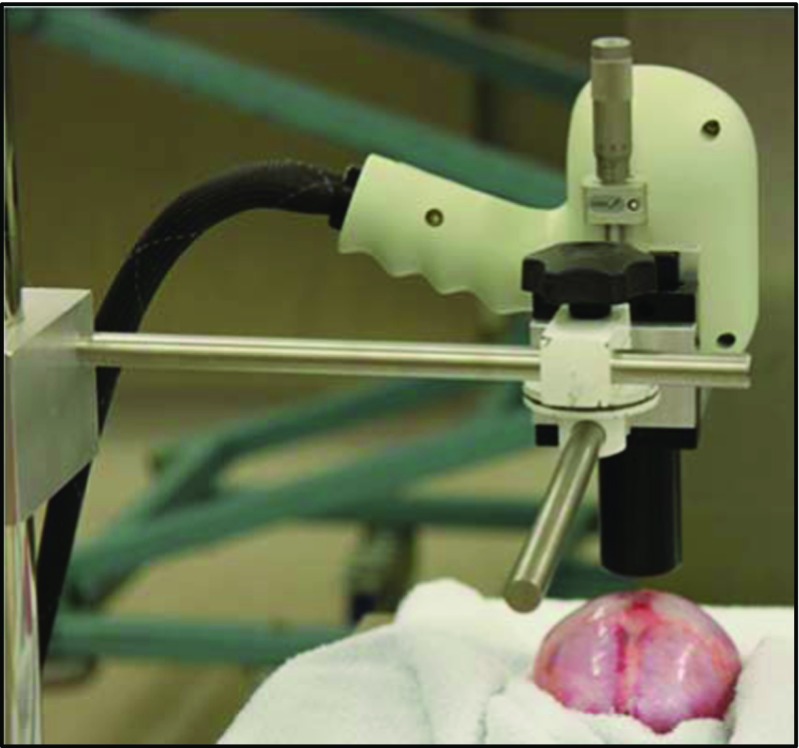
Stand for the benchtop OCT imaging probe

After removal of the calvarial bones, the dural membrane was optically cleared by a method previously described [30] to increase the depth of imaging. The imaging probe was secured at a working distance of 17 mm above the parietal dura by a stand specifically designed to secure the probe. A micrometer stage with *y*-axis movement allowed for fine adjustment of the probe to facilitate capture of 2-dimensional images of a small section of dura close to the SSS (Fig. 3). Two types of scanning mode were employed with the Bioptigen system: a line scan (or stic scan or zero pullback mode) and an area scan. For these proofs of concept experiments, the following areas of dura were scanned: (5 × 1) mm^2^, (5 × 5) mm^2^ and (10 × 10) mm^2^.

## Results

### Rotational optical coherence tomography

All accessible sections of the SSS were imaged. The catheter was also capable of imaging within the transverse sinus. However, the entire lumen was not captured in some areas of the transverse sinus, possibly due to the size of the lumen at those sites, but more likely owing to the curvature of this section of the sinus, making central positioning of the catheter challenging.

**Fig. 4:**
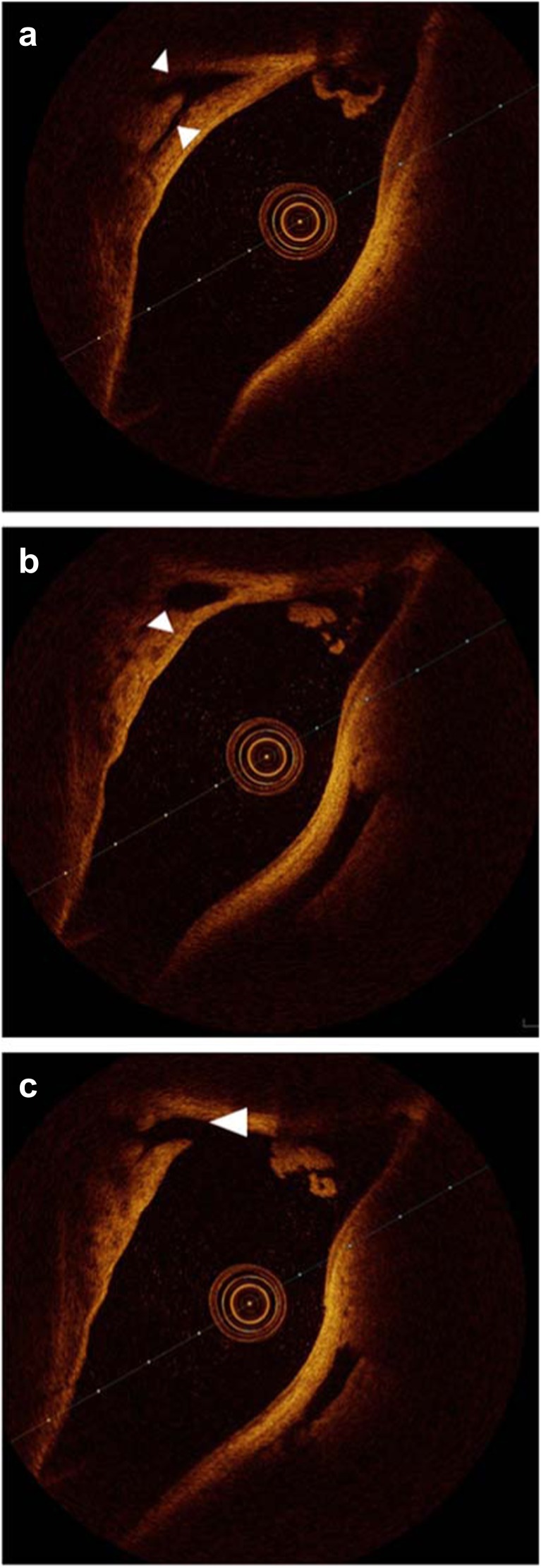
Sequential rotational OCT frames. **a** Branching vessels near the wall of the SSS (*arrow heads*). **b** Larger of the two branches just before joining the SSS (*arrow head*). **c** Vessel joining the SSS (*arrow head*)

**Fig. 5:**
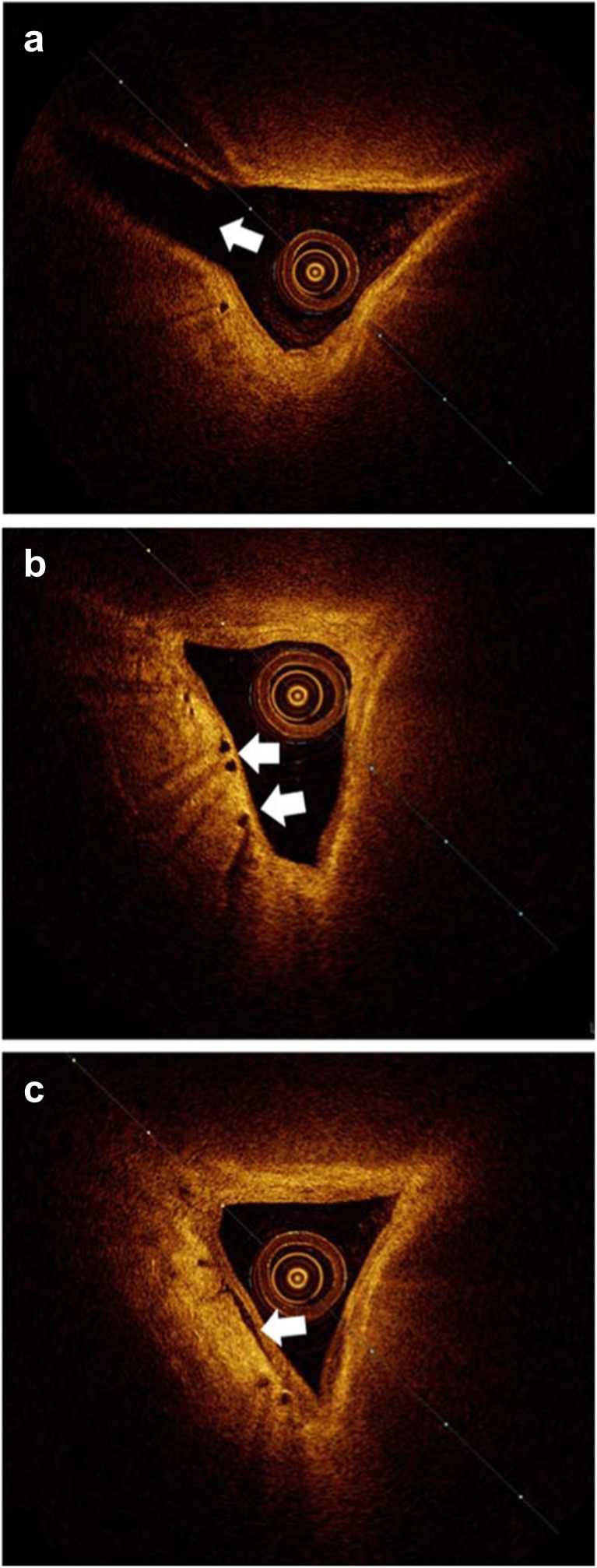
Vessel-like structures demonstrated with OCT. **a** Large blood vein joining the SSS (*arrow*). **b** Clusters of vessel-like structures in cross section (*arrows*). **c** Vessel-like structure in longitudinal section (*arrow*)

Numerous vessel-like structures were identified in both the anterior and posterior segments of the SSS. Confirmation that the structures were blood vessels could only be obtained when they were obviously seen to branch or were directly entering the SSS, indicating a role in blood drainage (Fig. 4). The relatively larger veins draining directly into the sinus often joined at a lateral apex of the triangular shaped sinus, whilst much smaller, vessel-like structures were seen clustering close to the walls of the sinus in both cross and longitudinal sections (Fig. 5).

Approximate diameter measurements were made of the small vessel-like structures close to the wall of the SSS when imaged in a cross-sectional plane, for both anterior and posterior segments of the sinus. The median inner diameters of the anterior and posterior segments were 80 μm (range 40 to 170 μm, *n* = 25) and 90 μm (range 30 to 150 μm, *n* = 23), respectively. Measurements were also made of gaps in the SSS wall where larger vessels appeared to be entering the sinus, providing approximate estimations of the size of these structures. The median gap measurements for the anterior and posterior segments were 110 μm (range 70 to 670 μm, *n* = 21) and 125 μm (range 70 to 740 μm, *n* = 23), respectively.

The mean cross-sectional area and diameter of the anterior section of the SSS were 2.65 mm^2^ (SD = 0.61) and 1.77 mm (SD = 0.21), respectively. The mean cross-sectional area and diameter of the posterior section of the SSS were 4.73 mm^2^ (SD = 1.76) and 2.34 mm (SD = 0.44), respectively.

### Benchtop optical coherence tomography

**Fig. 6:**
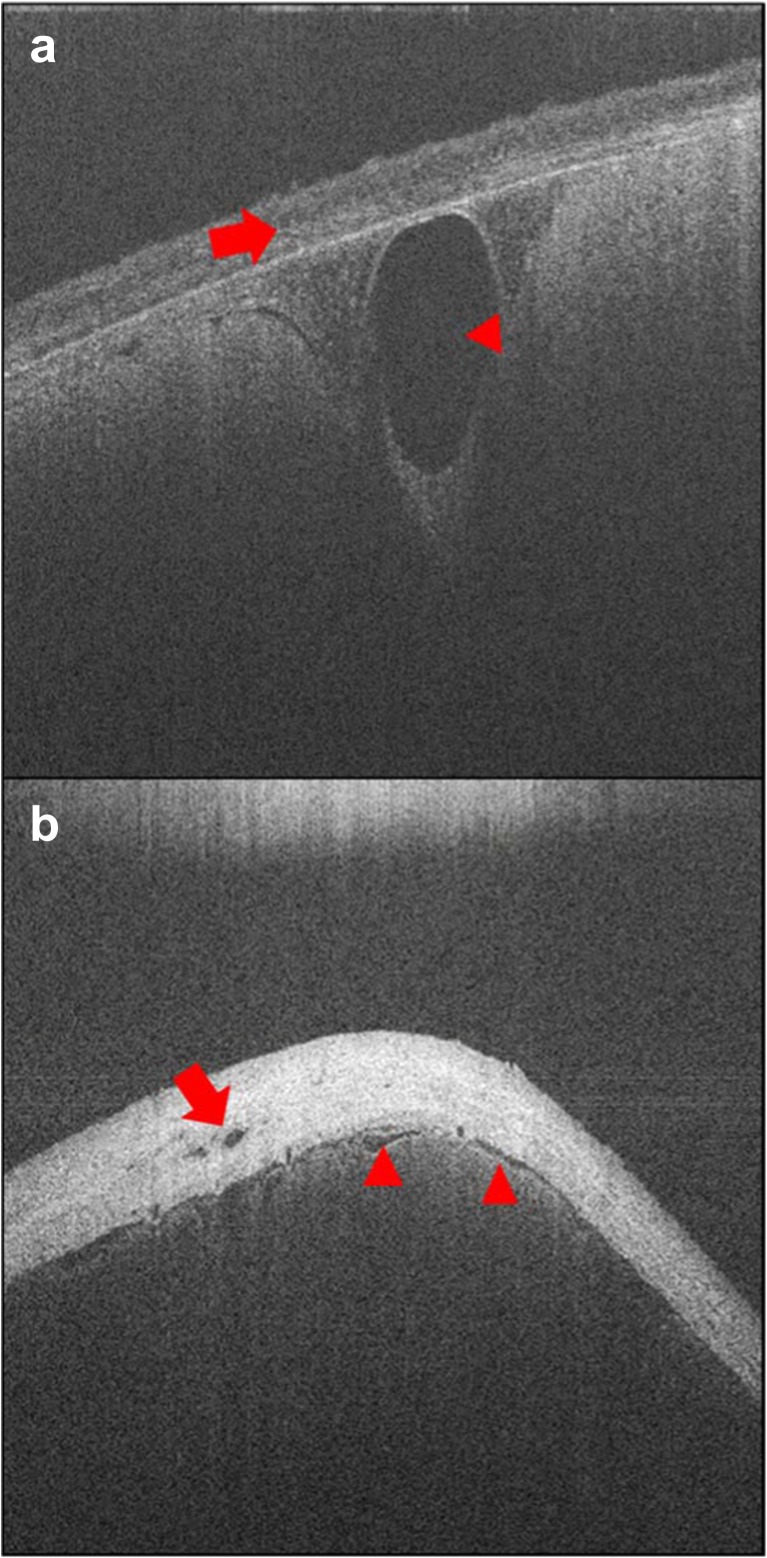
Two static images of the dural membrane using benchtop OCT. **a** Dura mater (*arrow*) and large probable lumen of a blood vessel just below the dural membrane (*arrow head*). **b** Vessel-like structures within the dura (*arrow*), and below the dura, in cross section and longitudinal (*arrow heads*)

Numerous vessel-like structures could be seen within and below the dural membrane, in both longitudinal and cross section, using the benchtop OCT. As with the rotational OCT system, confirmation of whether or not the structures were blood vessels could only be provided if they were seen to be branching. Several structures could be seen with relatively large lumina (larger than the depth of the dural layer), which were likely to be either bridging veins or cerebral veins (Fig. 6).

## Discussion

This paper demonstrates the potential for using post-mortem OCT for the high-resolution imaging of the blood vessels associated with the dura. With alternative hypotheses suggested for the source of SDH in AHT, detailed characterisation of the dural vasculature is of medicolegal as well as scientific importance.

Vessel-like structures appeared to be numerous and varied considerably in size, often appearing larger when directly entering the SSS. The estimated diameters of the veins entering the SSS are similar to the measurements in another small study on infant bridging veins [31]. The smaller vessels measured close to, or within, the wall of the SSS, and seen within the dura on the benchtop OCT system, were likely to have been dural blood vessels which have previously been described as a similar size to those imaged in this study [16].

Primary anastomotic arteries (PAA) branching from the main meningeal arteries are suggested to be approximately 100–300 μm in diameter and then branch further into secondary anastomotic arteries (SAA) which are around 20 to 40 μm diameter when located upon the endosteal surface of the dura [16]. Further SAA are stated as lying in the middle of the dura, being approximately 50 to 90 μm diameter, and penetrating vessels, which are most likely arterioles which extend deep into the dura close to arachnoid surface, are thought to be only 5 to 15 μm diameter. The arterioles are proposed to end in an extremely rich capillary network with vessels of approximately 8 to 12 μm which is most abundant parasagittally [16]. As the resolution capabilities of the systems used in this paper were around 20 μm, it is likely that the majority of the SAA and PAA vessels were imaged; however, it is possible that any extremely small capillary networks may require an imaging system with even higher resolution.

The measurements of the mean area and diameter of the anterior and posterior segments of the SSS agree with previous studies of the adult SSS which conclude that the size of the sinus increases from the frontal region to the occipital area. As would be expected, the diameter and cross-sectional area measurements of the SSS for the neonate in this study were smaller than those recorded for studies in the adult population [32].

In order to further validate OCT for the identification of small blood vessels within the dura, histological and immunohistochemical investigations would need to be undertaken alongside imaging studies to enable confirmation of the vascular nature of the identified structures.

Future development of this technique, particularly utilising the benchtop system, could enable detailed 3-dimensional reconstruction of the blood vessels within and directly below the dura for a larger area of the cerebral convexity and could also be used to image the falx and tentorium. Furthermore, in comparison to the rotational system, the benchtop system allows for a noninvasive examination of the structures of interest. We consider that this would require an automated computational system for sequential scanning of large areas and the collating of data through image mosaic and registration methods.

In conclusion, optical coherence tomography provides enhanced resolution capabilities when compared with other existing imaging modalities such as MR and CT, enabling detailed observation of very small sub-surface blood vessels.
